# Genome sequence of the halotolerant yeast *Debaryomyces hansenii* strain CM662

**DOI:** 10.1128/mra.01140-24

**Published:** 2025-05-19

**Authors:** Mariana Izaguirre-Beltrán, Cristian A. Flores-Cruz, Melissa Reynoso-Rodríguez, Saúl A. Martínez-Morales, Benito Pereyra-Alférez, Jorge H. García García

**Affiliations:** 1 Facultad de Ciencias Biológicas, Instituto de Biotecnología, Universidad Autónoma de Nuevo León, San Nicolás de los Garza, Nuevo León, Mexico; University of California Riverside, Riverside, California, USA

**Keywords:** biotechnology

## Abstract

We report the draft genome sequence of *Debaryomyces hansenii* strain CM662, isolated from the Sierra Madre Oriental in Nuevo León, Mexico. This strain exhibits antimicrobial activity against pathogenic bacteria such as *Escherichia coli* and *Listeria monocytogenes*. The genome assembly consists of 147 contigs totaling 12,144,590  bp, with a GC content of 36.04%.

## ANNOUNCEMENT

*Debaryomyces hansenii* is a halotolerant yeast species known for its ability to thrive in high-salt environments and its potential applications in biotechnology, particularly in food preservation and the production of bioactive compounds. Strain CM662 was isolated during a screening program aimed at discovering yeasts with antimicrobial properties from the Sierra Madre Oriental in Nuevo León, Mexico.

Sampling was conducted following adapted methodologies from Aljohani et al. (1) for soil and from Koricha et al. (2) and Into et al. (3) for vegetation. Four locations within the Sierra Madre Oriental were sampled over a period of slightly more than a year: La Piedra Parada in La Trinidad, Montemorelos (August 2020), La Cama de Piedra on Cerro de las Mitras, Monterrey (February 2021), route to Pico Norte on Cerro de la Silla (March 2021), and outskirts of Icamole in the municipality of García (October 2021).

At each site, coordinates, temperature, altitude, and vegetation type were recorded. Samples included leaves, bark, cladodes (in the case of Opuntia species), flowers, and fruits, depending on availability. Soil samples consisted of approximately 5 cm of the topsoil layer, with *in situ* pH measurements using Whatman indicator strips.

In total, 40 samples were collected from 27 different sources. *In situ* enrichment cultures were prepared by placing each sample into 50 mL of YM medium (yeast extract: 0.3%, peptone: 0.5%, malt extract: 0.3%, and glucose: 1%) supplemented with 0.02% chloramphenicol and 0.025% sodium propionate to inhibit bacterial and filamentous fungal growth, respectively. Cultures were transported to the laboratory for further processing.

Genomic DNA was extracted following the protocol described by Amery et al. (4). Briefly, 2 mL of a 24–48 h YM broth culture was grown in YM medium, centrifuged, and the cell pellet was resuspended in 400 µL of extraction buffer (200 mM Tris-HCl, pH 8.5, 25 mM EDTA, 250 mM NaCl, and 0.5% SDS). The mixture was incubated at 65°C for 15 minutes, followed by the addition of 130 µL of 3 M sodium acetate, pH 5.2. After vigorous vortexing and incubation at −20°C for 10 minutes, the sample was centrifuged at 13,000 rpm for 15 minutes at 4°C. The supernatant was incubated with an equal volume of isopropanol at −20°C for 20  minutes. DNA was pelleted by centrifugation at 6,000 rpm for 20 minutes at 4°C, washed twice with absolute ethanol and once with 70% ethanol, and then centrifuged at 7,000 rpm for 5 minutes. The DNA pellet was resuspended in TE buffer (10 mM Tris-HCl pH 8 and 1 mM EDTA) and quantified using a NanoDrop 2000 spectrophotometer (ThermoFisher).

Whole-genome sequencing was performed by GeneWiz using the Illumina MiSeq platform with the Illumina Nextera XT Library Preparation Kit, generating a paired-end run of 250 bp reads, yielding 21 million reads of raw data, with a mean quality score of 35.30 (10,784 Mb, 875× coverage). A paired-end library with 300 bp reads was constructed. Quality assessment of raw reads was conducted using FastQC version 0.11.9 (5). Adapter sequences and low-quality bases were trimmed using TrimGalore version 0.6.6 (6).

*De novo* genome assembly was performed using SPAdes version 3.15.3 (7), with default k-mer set as 25, 33, 55, and 127 (7). The assembly resulted in 147 contigs larger than 1,000 bp, with a total length of 12,144,590 bp and an *N*_50_ value of 672,275 bp. The genome completeness was evaluated with BUSCO version 5.8.2 using the saccharomycetes_odb10 database (8). The largest contig was 1,604,087 bp, and the GC content was 36.04% (Table 1). Assembly statistics were evaluated using QUAST version 5.0.2 (9).

**TABLE 1:** Summary of sequencing and assembly metrics for *Debaryomyces hansenii* strain CM662^*a*^

Metric	Value
Reads (bp)	21,567,708
Yield (Mb)	10,784
Mean quality Phred	35.30
Coverage	875×
Contigs	147
Number of contigs (≥50,000 bp)	29
Largest contig (bp)	1,604,087
Total length	12,144,590
*L*_50_	7
GC (%)	36.04
BUSCO complete	99.6%
BUSCO single copy	99.4%
BUSCO duplicated	0.1%
BUSCO missing	0.4%
BUSCO error (internal stop codon)	0.1%
BUSCO groups searched	2,137

^*a*^ The table includes read count, total bases (Mb), mean Phred quality, estimated coverage, number of contigs, and assembly quality indices (*N*_50_, *L*_50_, and GC content) and BUSCO completeness metrics using the saccharomycetes_odb10 database.

## Data Availability

The whole-genome shotgun project has been deposited at GenBank under accession number JBGOPX000000000 (BioProject number PRJNA1150158 and BioSample number SAMN43272277). Raw sequencing data are available in the Sequence Read Archive (SRA) under accession number SRR30343427.

## References

[1] Aljohani MD, Abdulmajeed AM, Fatani KA, Gherbawy YA, Bahkali AH. 2018. Isolation and characterization of soil yeasts from western region of Saudi Arabia. Saudi J Biol Sci 25:1678–1683. doi:10.1016/j.sjbs.2016.09.01130591785

[2] Koricha T, Alebachew T, Woyessa D. 2019. Isolation and characterization of yeast species from fruits and fermented beverages in Ethiopia. Int J Microbiol 2019:9567932. doi:10.1155/2019/9567932

[3] Into P, Jatuwong K, Saichana N. 2020. Diversity and distribution of yeast communities in the phyllosphere of rice plants grown under organic and conventional farming systems. Microorganisms 8:1581. doi:10.3390/microorganisms8101581 33066632 PMC7602415

[4] Amery V, Trezzi JP, Lievens B, Honnay O. 2011. Simple and effective DNA extraction protocol for the fungal endophyte Piriformospora indica. Lett Appl Microbiol 52:75–81. doi:10.1111/j.1472-765X.2010.02969.x

[5] Andrews S. 2010. FastQC: a quality control tool for high throughput sequence data. Available from: https://www.bioinformatics.babraham.ac.uk/projects/fastqc

[6] Krueger F. 2023. TrimGalore: adaptive quality and adapter trimming. Available from: https://www.bioinformatics.babraham.ac.uk/projects/trim_galore

[7] Cha S, Bird DM. 2016. Optimizing k-mer size using a variant grid search to enhance de novo genome assembly. Bioinformation 12:36–40. doi:10.6026/97320630012036 28104957 PMC5237644

[8] Manni M, Berkeley MR, Seppey M, Simao FA, Zdobnov EM. 2021. BUSCO update: novel and streamlined workflows along with broader and deeper phylogenetic coverage for scoring of eukaryotic, prokaryotic, and viral genomes. Available from: http://arxiv.org/abs/2106.11799 10.1093/molbev/msab199 PMC8476166 34320186

[9] Prjibelski AD, Antipov D, Meleshko D, Lapidus A, Korobeynikov A. 2020. Using SPAdes de novo assembler. Curr Protoc Bioinform 70.doi:10.1002/cpbi.102. 32559359

